# Quality Attributes of Cold-Stored Khalal Barhi Dates Treated with Guava Leaf Extract and/or Lactic Acid as Natural Preservatives

**DOI:** 10.3390/foods12112115

**Published:** 2023-05-24

**Authors:** Nashi K. Alqahtani, Tareq M. Alnemr, Fahad Al-Asmari, Salim A. Ali

**Affiliations:** 1 Department of Food and Nutrition Sciences, College of Agricultural and Food Sciences, King Faisal University, Al-Ahsa 31982, Saudi Arabia; talnemr@kfu.edu.sa (T.M.A.); falasmari@kfu.edu.sa (F.A.-A.); 2 Date Palm Research Center of Excellence, King Faisal University, P.O. Box 400, Al-Ahsa 31982, Saudi Arabia; 3 Department of Food Science, Faculty of Agriculture (Saba Basha), Alexandria University, Alexandria 21531, Egypt

**Keywords:** Barhi dates, guava leaf extract, lactic acid, physicochemical properties, antioxidant activity, sensory evaluation, microbiological quality

## Abstract

The use of natural ingredients to preserve the quality of fresh fruits is a promising approach to healthier products and a more sustainable industry. The present study was carried out to assess the effect of lactic acid (LA) and guava leaf extract (GLE) as natural preservatives on the quality parameters of Khalal Barhi dates. Physicochemical properties, antioxidant activity, color parameters, firmness, sensory properties, and yeast and mold counts of date fruits were evaluated during five weeks of storage at 4 ± 1 °C. The bioactive compounds in GLE were estimated by HPLC, which exhibited that GLE contains significant amounts of bioactive compounds, mainly, phenolics and flavonoids. With prolonged storage, the moisture content decreased, while the total soluble solids (TSS) increased in all samples. Similarly, a slight decrease in the pH with a concomitant increase in titratable acidity (TA) was observed throughout the storage. Generally, the samples treated with natural preservatives revealed lower changes in moisture content, TSS, pH, and TA than the control. The results exhibited decreased total phenolic content (TPC) and antioxidant activity for all samples with extended storage. The GLE and LA + GLE treatments significantly (*p* < 0.05) increased TPC and antioxidant activity on day 0 and preserved higher values of both during storage. Additionally, a decrease in the L* and b* values with an increase in the a* values of all samples was observed with advancement of storage. The LA + GLE treatment minimized the changes in color parameters and maintained higher firmness values during storage. Similarly, the sensory properties of all samples decreased with prolonged storage, but insignificant (*p* > 0.05) differences were found among the samples. Dipping treatments inhibited microbial growth over time, with the lowest yeast and mold counts achieved by the LA + GLE treatment. It can be concluded that the LA + GLE treatment has a protective effect on Khalal Barhi dates by minimizing post-harvest changes and decreasing the microbial load.

## 1. Introduction

In several countries, date palm (*Phoenix dactylifera*) represents the most important fruit crop [1]. After Egypt, Saudi Arabia is the second largest date palm producer, with an annual production of 1.57 million tonnes [2]. Several reports confirmed the great importance of date palm due to its great nutritional value, multiple health benefits, and its role in agroecosystems in addition to supporting the national economy [3]. Barhi, one of the most popular date varieties in the Arabian Peninsula, is commonly consumed at Khalal stage. Generally, Barhi date fruits pass through four stages during the ripening process, including: (1) the immature and nonedible date fruits with full green color and rigid texture, which are known as Kimri; (2) the partially ripe fruits with yellow color, crispy texture, and delicious taste, which are called Khalal; (3) the soft and semi-ripe fruits with light brown color, chewy texture, and a caramel-sweet taste, which are known as Rutab; and (4) the softer and fully ripe date fruits with dark brown color, which are called Tamer [4]. The higher consumer demand for this variety is mainly correlated to its distinct organoleptic qualities and related health advantages due to the presence of flavor components and phytochemicals, particularly phenolics and flavonoids [5]. However, Barhi date fruits are rapidly converted from Khalal to Rutab if they are not preserved properly. Accordingly, finding effective and suitable methods to keep Khalal Barhi dates’ quality after harvest and throughout the storage, handling, and marketing for a longer time is of vital importance.

Various methods have been applied to maintain fruit quality during storage, e.g., chemical preservatives, modified atmosphere packaging, controlled atmosphere storage, UV light, electron beams, ozone, chlorine dioxide, and hydrogen peroxide treatment [4]. In recent years, more attention has been paid to the application of natural preservatives, particularly plant extracts, in the food industry [6]. Such preservatives improve the functionality of food products, develop wellness foods, and produce healthier products. *Psidium guajava*, a fruit crop commonly known as guava, is cultivated in all tropical and subtropical countries [7]. Guava leaf extract (GLE) is a good source of polyphenolics, e.g., ellagic acid, gallic acid, caffeic acid, chlorogenic acid, p-coumaric acid, rosmarinic acid, apigenin, catechin, hyperin, myricetin, guaijaverin, kaempferol, naringenin, naringin, rutin, and quercetin [8,9,10]. Accordingly, numerous researchers confirmed the antimicrobial and antioxidant properties of GLE [6,7,8,9,10,11,12]. In the guava plantation, significant amounts of guava leaves are discarded as agricultural waste through the pruning process every year after the fruit harvest, which can increase environmental pollution. Generally, in the last few years, more efforts have been made for upcycling of agricultural by-products to develop value-added products in the food and pharmaceutical industries. Such an orientation may reduce environmental pollution by minimizing the amounts of agricultural waste, convert low-value products to high-value ones, produce new food varieties, and therefore, improve the local and international economies, which can be consistent with the UN’s goals for sustainable development [13].

Lactic acid (LA) or 2-hydroxypropionic acid, generally recognized as a safe (GRAS) substance according to the FDA, is one of the high-volume chemicals produced microbially through industrial fermentation processes using lactic acid bacteria in the genus *Lactobacillus* and molds in the genus *Rhizopus*, and is used in the food, pharmaceutical, and chemical industries [14]. LA is a strong antimicrobial agent against a wide range of pathogenic and food spoilage microorganisms; accordingly, it can be used as an efficient food preservative [15,16,17,18]. In addition, previous reports confirmed the disinfection effects of LA in different food products, e.g., apples [16], oranges, tomatoes [17], broccoli [18], cantaloupes, bell peppers [19], lettuce [20], meat, and poultry products [21]. Therefore, we used LA in the current study to improve the preservation efficiency of GLE.

The aim of the current investigation was to assess the impact of GLE and/or LA on the physical, chemical, and microbiological qualities of fresh Khalal Barhi dates throughout the storage period.

## 2. Materials and Methods

### 2.1. Materials

Fresh Khalal Barhi dates and fresh guava leaves were procured from the farm of Research and Training Station, King Faisal University, Al-Ahsa, Kingdom of Saudi Arabia. The fruit trees were grown in tropical climate and subjected to model horticultural practices recommended by a staff member of the university. For the main experiment, dates without physical damage, insect bites, and microbial spoilage were used. Sigma–Aldrich (Sigma–Aldrich Chemie GmbH, Steinheim, Germany) supplied chemicals and reagents (sodium hydroxide, phenolphthalein indicator, methanol, acetone, Folin–Ciocalteu reagent, sodium carbonate, lactic acid, DPPH solution, gallic acid, Rose-Bengal chloramphenicol medium), whereas LDPE bags were procured from the local market.

### 2.2. Preparation of Guava Leaf Extract (GLE)

The guava leaves were washed carefully and dried in a hot air oven at 50 °C for 24 h till the moisture content became < 8% (the recommended value for producing and storing guava leaf powder) [9,22]. Subsequently, the dried leaves were ground using a kitchen grinder and stored at 4 °C until extraction. The extraction was carried out following the method described previously [7,23]. Briefly, 100 g of guava leaf powder was added to 1 L distilled water (d.w), stirred for 4 h at 70 °C, cooled at room temperature, and the mixture was filtered using Whatman filter paper No. 4 to remove impurities. To obtain the maximum extracted yield, the residue was further extracted with 200 mL of d.w. The extract was collected and lyophilized in a freeze drier. The dried extract was powdered and stored at 4 °C for further use.

### 2.3. HPLC Analysis of GLE

Characterization and quantification of phenolics and flavonoids in GLE were carried out by HPLC (Agilent 1260, Agilent Technologies, Santa Clara, CA, USA) with a VL quaternary pump, UV/Vis detector, and C18 column (250 × 4.6 mm, 5 μm particle size). The operation conditions applied for separation and identification are mentioned in our previous work [4]. The chromatogram (Figure S1) was obtained and analyzed by Agilent ChemStation. Standards’ quantification is mentioned in the supplementary material (Table S1). Measurements were conducted in duplicate, and outcomes were given as mg/g of GLE.

### 2.4. Treatment of the Fresh Date Fruits

The immersion solutions (LA (1%), GLE (0.1%), LA (1%) + GLE (0.1%), and the control (d.w)) were used to disinfect fresh date fruits by soaking for 5 min according to our previously published study [16]. Then, to remove moisture, the treated fruits were spread on a clean stainless-steel table for 3 h at room temperature. Subsequently, the samples were put in LDPE bags (250 g ≈ 13 fruits), with a total of 6 bags for each treatment, and kept at 4 ± 1 °C with 80% RH [24]. Samples were withdrawn at the end of each week of storage for further analyses.

### 2.5. Shelf-Life Studies of the Treated Date Fruits

#### 2.5.1. Physicochemical Qualities

For date samples, the moisture content was estimated gravimetrically with a direct oven-drying method. Briefly, 10 g of the samples was dried at 105 °C till a constant weight, and the moisture content (%) was calculated as follows: [(wet weight − dry weight)/wet weight] × 100 [16]. The TSS content was estimated with an N-50E ATAGO^®^ refractometer at 25 °C. A few drops of the samples’ juice were placed on the clean angled prism using a pipette, the clear cover was sealed, and the readings (°Brix) were taken directly through the eyepiece by pointing the refractometer at a direct light. The date fruits’ pH was determined in the slurry obtained by mixing 10 g of the sample in 20 mL of d.w for 2 min using AD1030, ADWA^®^ pH meter. The TA values of the samples’ slurry were estimated using the titration method according to the procedures described earlier [5,16] using NaOH solution (0.1 N) and phenolphthalein as an indicator. The TA values were given as % of malic acid/100 g sample.

#### 2.5.2. Total Phenolic Content (TPC)

The method described by Alqahtani et al. [1] was followed to extract the phenolic compounds from the samples. Ten grams of date samples were mixed with 40 mL of methanol–water (50:50, *v*/*v*), agitated at 60 °C for 1 h using a magnetic stirrer, and centrifuged for 10 min at 4000× *g*. The supernatant was collected, and the residue was further extracted with 40 mL of acetone–water (70:30, *v*/*v*), agitated at 60 °C for 1 h, centrifuged, and the supernatant was collected. Both supernatants were mixed, and the volume was completed to 100 mL with d.w for TPC and antioxidant estimations. The TPC of date samples was determined according to Corrêa et al. [25]. Briefly, 5 mL of d.w, 0.5 mL of Folin–Ciocalteu reagent, and 1.5 mL of Na_2_CO_3_ (15%) were mixed with 0.1 mL of samples’ extracts, and the mixture was completed to 10 mL using d.w and incubated for 2 h at room temperature in the dark. The absorbance was estimated with a UV/VIS JENWAY^®^ spectrophotometer at 765 nm, and the results were given as mg GAE/g sample.

#### 2.5.3. Antioxidant Activity

Using the DPPH method, the dates’ antioxidant activity was assessed according to the method described previously [5]. One mL of the samples’ extract was mixed with 2 mL of DPPH solution and kept for 30 min at room temperature. The absorbance values of the mixtures were determined with a spectrophotometer at 517 nm, and the results were given as the DPPH scavenging activity percentage.

#### 2.5.4. Color Parameters

The date fruits’ color parameters were measured with a HunterLab colorimeter (MiniScan^®^ EZ 4500L, HunterLab, Reston, VA, USA) according to the method outlined by Alqahtani et al. [4]. The device was calibrated with a black and white tile, the readings were taken from three different places on the fruit’s surface, and the average was calculated. The measured color parameters were L* (0 = black, 100 = white), a* (−60 = greenness, +60 = redness), and b* (−60 = blueness, +60 = yellowness).

#### 2.5.5. Firmness

Firmness was measured with a Stable Micro Systems texture analyzer according to the method described by Abdelkarim et al. [26]. Measurements were performed by a cylindrical probe at the following conditions: 5 mm depth, 1.5 mm/s velocity, and 25 °C temperature.

#### 2.5.6. Sensory Properties

A sensory evaluation of freshly treated and stored Barhi date fruits was conducted by 10 semi-trained panelists. According to the method described by Alhamdan et al. [27], the appearance, odor, taste, texture, and overall acceptability of the samples were evaluated on the first day of treatment and at the end of each week of storage using a 9-point hedonic scale (1 = extremely dislike, 9 = extremely like).

#### 2.5.7. Microbiological Analysis

According to Voon et al. [28], the spread plate technique was followed to monitor yeast and mold growth in treated and untreated Barhi date samples using Rose-Bengal chloramphenicol (RBC) medium. Briefly, 25 g of date samples was mixed in a Stomacher blender for 1 min with 225 mL of a sterile saline solution. Subsequently, 0.1 mL of the diluted sample was spread on the surface of a solid RBC medium. The plates were incubated for 3–5 days at 25 °C, and the readings were taken and expressed as log CFU/g.

### 2.6. Statistical Analysis

Measurements were made in triplicate and expressed as means ± standard deviation. Significant differences (*p* < 0.05) between treatments were analyzed with two-way analysis of variance (ANOVA) using SPSS statistical software (SPSS Inc., version 16.0, Chicago, IL, USA) [4].

## 3. Results and Discussion

### 3.1. HPLC Analysis of GLE

Remarkable amounts of polyphenols, mainly phenolics and flavonoids, were detected through the HPLC analyses of GLE (Table 1 and Figure S1). In general, ellagic acid, gallic acid, and pyrocatechol were the three major phenolic compounds found in GLE, with concentrations of 18.40, 14.86, and 7.59 mg/g, respectively. Moreover, seven phenolic compounds in lower concentrations were also detected, namely ferulic acid, vanillin, chlorogenic acid, caffeic acid, methyl gallate, syringic acid, and cinnamic acid. Similarly, the results revealed that naringenin and catechin were the two main flavonoids detected by HPLC analysis of GLE, with amounts of 2.48 and 0.90 mg/g, respectively. In addition, two flavonoids were also detected in low concentrations, namely quercetin and rutin. Our results are consistent with those reported by Ruksiriwanich et al. [9], who found that GLE contains the following polyphenols: rutin, quercetin, naringenin, catechin, epicatechin, gallic acid, kaempferol, and guaijaverin. In addition, Sampath Kumar et al. [29] showed that GLE contains bioactive compounds such as ellagic acid, gallic acid, quercetin, esculin, gallocatechin, 3-sinapoylquinic acid, and citric acid. In fact, the differences in the HPLC results for GLE in some reports could be due to the variations in the applied extraction techniques, guava type, soil quality, climate conditions, duration of exposure to sunlight, and agricultural practices. It is worth mentioning that the bioactive compounds present in GLE, particularly phenolics (e.g., ellagic acid, gallic acid, and pyrocatechol) and flavonoids (e.g., naringenin and catechin), are responsible for its unique antioxidant activity [9,29], which is in line with the results for TPC and antioxidant activity in the current study (Section 3.3).

### 3.2. Physicochemical Qualities

The effects of natural preservatives on the moisture content, TSS, pH, and TA of freshly treated and cold-stored (4 ± 1 °C) Barhi dates are displayed in Table 2. In fact, the loss of moisture is a crucial parameter of fruit quality deterioration; hence, it should be monitored throughout the storage. In general, the moisture content of all date samples decreased significantly (*p* < 0.05) as storage progressed, from 68.38–68.44 to 62.21–63.04%. However, all samples retained a moisture content higher than 50% up to the end of storage. Ghafoor et al. [5] attributed the moisture loss of the dates during storage to the conversion from the Kimri to Rutab stage. Furthermore, Seddiek et al. [16] attributed the loss of water in fruits during storage to the catabolic process that occurred naturally during respiration and senescence. In general, samples treated with LA, GLE, and LA + GLE revealed less moisture loss compared to the control. Furthermore, the LA + GLE treatment decreased the moisture loss compared to other treatments, and this was evident after 3 weeks. Khan et al. [12] reported that GLE contains several classes of compounds, such as steroids, triterpenoids, lignans, anthocyanins, organic acids, phenylpropanoids, phenolics and flavonoids. It could be suggested that such compounds may form a thin film coating on the dates’ surface that may partially act as a barrier against gases and vapors and, therefore, can prevent moisture loss, decrease the respiration rate, and delay ripening and senescence processes. Furthermore, LA may decrease enzymatic activity, which in turn may decrease respiration, senescence, and other metabolic processes, resulting in less moisture loss [16]. Our results are consistent with the reports of Kumar et al. [30] and Seddiek et al. [16], who found that the pomegranate peel extract-based film and LA + natural plant extracts prevented green bell pepper and apple fruits, respectively, from losing weight during storage.

The TSS concentration, an important parameter that measures fruit ripening and maturity, of all samples increased (*p* < 0.05) with extended storage, from 27.15–27.19 to 32.02–33.77% (Table 2). Similarly, the increase in TSS with extended storage period was recorded by Abu-Shama et al. [31] and Abdelkarim et al. [26] for date fruits. Gull et al. [32] attributed the increase in TSS values with progressed storage to the degradation of complex polysaccharides into simple sugars. Moreover, Abebe et al. [33] ascribed the increase in TSS to the transformation of some compounds from an insoluble to a soluble form, such as protopectin into pectin, and the increase in fruits’ juice concentration to the moisture loss. Among the treatments, the highest increase in TSS content was recorded at the end of storage time in control fruits (33.77%), while the LA + GLE treatment showed the lowest (32.02%). However, LA + GLE treatment could be expected to decrease respiration rate, retard degradation of polysaccharides, and consequently, decrease the dates’ TSS changes throughout storage. The present results are in agreement with the study conducted by Seddiek et al. [16] who observed that treatment with LA + natural plant extracts decreased the changes in TSS content of apple fruits during cold storage.

The results in Table 2 exhibited a decrease in the pH values (from 6.07–6.25 to 4.93–5.21) with a concomitant increase in TA (from 0.13–0.15 to 0.24–0.28%) with extended storage. Tabikha et al. [34] illustrated the decrease in the pH of stored fruits with the metabolic activity of microorganisms that can convert some sugars into alcohols and acids. Additionally, the decrease in the pH values of the stored dates could be explained by the natural changes that occurred with the progression of the maturity process; that is, the pH can reach its lowest value in the ripening stage [35]. However, on the first day of treatment, LA and LA + GLE treatments revealed lower pH values (6.07 and 6.17, respectively) with higher TA values (0.15 and 0.14%, respectively) compared to other treatments, which could be related to the impact of acid in disinfection solutions.

Generally, throughout storage, LA, GLE, and LA + GLE treatments exhibited better storage stability in terms of pH and TA values than the control. Moreover, the LA + GLE treatment preserved pH and TA values compared to other treatments, with pH and TA values of 5.21 and 0.24%, respectively, at the end of storage (5 weeks). It could be expected that the combined effect of LA + GLE may decrease respiration rate, delay catabolic processes, slow enzymes activity, and consequently, delay fruits’ ripening, which could preserve pH and TA values throughout storage. However, the results of the present study were in agreement with the reports of Kumar et al. [30] and Alqahtani et al. [4], who recorded that pH decreased and TA rose with prolonged storage in green bell peppers and Khalal Barhi dates. They reported that the treatment with natural extracts decreased the changes in pH and acidity with storage.

### 3.3. TPC and Antioxidant Activity

The data presented in Table 3 exhibited a significant difference (*p* < 0.05) in the TPC of treated and untreated Barhi dates during storage. Freshly treated samples (GLE and LA + GLE) showed higher TPC values compared to control and LA, which was mainly related to the presence of remarkable amounts of phenolics in the GLE and LA + GLE disinfection solutions, as GLE has a higher content of bioactive compounds [8]. For all samples, TPC decreased dramatically (*p* < 0.05), from 8.18–9.91 to 3.27–4.42 mg GAE/g with the progress of storage. Likewise, the decline in TPC with prolonged storage time was observed by Alqahtani et al. [4] and Tappi et al. [36] for cold-stored date and apple fruits, respectively. In fact, Kumar et al. [30] attributed the drop in TPC with storage to the decomposition of polyphenolics by enzymes, for example, PPO and POD. In general, among all samples, Barhi dates treated with LA + GLE exhibited higher TPC than GLE, LA and control with 4.42, 4.35, 4.07, and 3.27 mg GAE/g, respectively, at the end of the experiment. It could be suggested that the synergistic effect of LA and the natural antioxidants present in GLE can decrease enzymatic activity, slow oxidation reactions, reduce ethylene production, and consequently prevent the degradation of polyphenolics of treated samples with prolonged storage [4]. Our findings are in harmony with those recorded by Tappi et al. [36] and Ghafoor et al. [5] for apple and Khalal Barhi dates, respectively.

Similarly, GLE and LA + GLE-treated samples showed higher antioxidant activity (48.18% and 47.82%, respectively) after treatment and before storage compared to control (44.28%) and LA (44.29%), indicating that GLE enhanced the antioxidant activity of the treated dates. Consistent with the TPC results of the current study, the antioxidant activity exhibited a gradual decrease (*p* < 0.05) with progressed storage time (from 44.28–48.18 to 23.76–26.87%). The decrease in antioxidant activity could be attributed to the drop in fruits’ phenolic content due to senescence, higher respiration rates, or the enzymatic oxidation of phenolic compounds by PPO and POD with increasing storage time [4,32]. However, control displayed the lowest antioxidant activity (23.76%), whereas LA + GLE treatment showed the highest (26.87%) after 3 and 5 weeks, respectively. Generally, the antioxidant activity against oxidative damage is usually due to the presence of polyphenolic compounds. Consequently, GLE may prevent the oxidative destruction of bioactive compounds of treated Barhi dates, which could preserve higher DPPH values during the storage period. Additionally, the combined effect of LA + GLE may improve the storage stability and minimize the decline in TPC and DPPH activity in Barhi dates compared to other samples. It is worth mentioning that the antioxidants in the GLE may protect fruit from multiple oxidation reactions, reduce tissue damage, prevent the loss of nutritional quality, decrease microbial growth, enhance several physiological and metabolic processes, and improve health benefits [8]. The present results were in agreement with previous findings reported by Gull et al. [32] and Alqahtani et al. [4], who recorded a decrease in antioxidant activity in apricot fruits and Barhi dates during cold storage, respectively. These researchers emphasized that the antioxidant activity could be preserved during storage by using natural extracts.

### 3.4. Color Parameters

Color is a crucial quality parameter that dramatically influences consumers’ choice of a food product. Changes in color parameters (L* (lightness or brightness: 0 = black, 100 = white), a* (−60 = greenness, +60 = redness) and b* (−60 = blueness, +60 = yellowness)) of control and treated Barhi dates during storage are mentioned in Table 4. A significant decrease (*p* < 0.05) from 54.15–55.83 to 41.56–42.88 in the L* value of all Barhi date samples was observed during storage. In addition, it could be noted that the control displayed a greater decrease in L* value compared to other samples. Ghafoor et al. [5] attributed the decrease in the L* value during storage to the enzymatic activity, mainly of catalase, PPO, and POD, which can result in discoloration of stored dates. Additionally, Siddiq et al. [37] attributed the changes in date fruits’ color with prolonged storage to the natural ripening process, which includes transformation of sucrose into reducing sugars and decomposition of fruits’ native pigments, resulting in the transition of dates from the Khalal to Rutab stage. Consistent with our results, Alqahtani et al. [4] and Gull et al. [32] recorded a decrease in the date and apricot fruits’ L* values, respectively, during storage.

The results displayed in Table 4 showed that the a* values of the control and treated samples rose gradually (*p* < 0.05), from 1.32–1.44 to 2.00–2.13 with the prolonged storage, revealing a decline in the dates’ greenness. Generally, control showed the highest increase (2.13) in a* value, while LA + GLE treatment exhibited the lowest (2.00) after 3 and 5 weeks of storage, respectively. Matching with our results, Atia et al. [24] reported that Barhi dates’ a* value rose with storage. However, the increase in the a* value during storage could be attributed to the decomposition of native pigments by enzymes, mainly chlorophyll, through the ripening processes of dates [4]. Regarding the value of b*, the results presented in Table 4 showed a gradual decrease (*p* < 0.05) in the value of b* (from 35.14–36.71 to 22.05–23.87) for all Barhi date samples throughout storage, revealing a drop in the yellowness of the fruits, but the control showed a greater decrease than the treated samples. Alqahtani et al. [4] illustrated the drop in fruits’ b* value with storage by the hydrolysis of carotenoid pigments, the production of brown pigments, and non-enzymatic Maillard browning. Our observations are consistent with the reports of Ghafoor et al. [5], who recorded a drop in Barhi dates’ b* value with storage.

Generally, LA + GLE treatment revealed the highest storage stability in terms of color attributes, whereas control exhibited the lowest. It could be suggested that the combined effect of LA and the antioxidants present in natural plant extracts may retard browning reactions, decrease respiration rates, reduce catabolic processes, slow the ripening of fruits and prevent the degradation of native pigments, which can minimize color changes in stored fruits [16,30,38]. However, changes in the dates’ color in the current research were consistent with previous reports [4,5]. These authors observed that L* and b* values dropped, while a* values rose throughout the cold storage of Khalal Barhi dates. Furthermore, they confirmed that the natural extracts of pomegranate and orange peel minimized the color changes in Barhi dates, respectively.

### 3.5. Firmness

Firmness or hardness is a critical factor that reflects the quality during storage. It is defined as the resistance of the food to deformation and is equal to the force needed to break the food between molars [4]. Table 5 shows the effects of disinfection treatments on the hardness of cold-stored Khalal Barhi dates. The hardness of all samples was reduced (*p* < 0.05) with extended storage, revealing a gradual drop in the quality of the fruits. The control sample showed the greatest decrease in firmness, while the LA + GLE treatment revealed the least. Our findings are consistent with those recorded by Zheng et al. [39] and Gull et al. [32], who observed a decrease in the firmness of apple and apricot fruits with extended storage, respectively. However, fruit softening (loss of strength) occurs due to the decomposition of the cell wall and degradation of the cell structure, resulting in the deterioration of the texture of the fruits, which in turn leads to a lower consumer demand for the product [38]. In addition, Kumar et al. [30] ascribed the drop in fruit firmness throughout storage to lipid oxidation, pectin component degradation, and moisture loss during transpiration. Furthermore, Gull et al. [32] attributed the loss of firmness to the reduction in cell wall mechanical strength throughout the ripening process by enzymatic activity, mainly of pectin methylesterase, galactosidase, and polygalacturonase. However, the LA + GLE treated sample showed higher firmness than other samples throughout the storage period, which can be illustrated by the fact that LA + GLE can decrease the respiration rate, delay catabolic activity, slow the ripening of fruits, maintain cell turgor by reducing the hydrolytic enzymes’ activity, and consequently, preserve higher firmness values. The findings of the current investigation are consistent with a previous study carried out by Kumar et al. [30] and Yang et al. [40], who found that pomegranate peel extract and blueberry leaf extract preserved the texture properties of green bell pepper and blueberries during cold storage.

### 3.6. Sensory Evaluation

In the current research, sensory evaluation was carried out to predict consumers’ acceptability and perception of treated Khalal Barhi date fruits. The changes in sensory evaluation parameters of dates throughout storage are displayed in Table 6. Generally, all samples exhibited a drop in appearance scores during storage, which could be due to the loss of moisture content, degradation of natural pigments, and progression of senescence [37]. Additionally, dates treated with LA + GLE showed higher appearance scores compared to other treatments up to the end of the experiment. However, at the end of storage, the greatest reduction in appearance scores (6.17) was recorded by control, while LA + GLE treatment revealed the least (6.84). It should be noted that LA treatment revealed lower appearance scores than GLE and LA + GLE throughout the storage period, which could be illustrated by the effect of LA on the pigments and tissues.

Similarly, a decrease in the odor and taste scores for all samples was observed during storage, with the greatest drop achieved by control, whereas LA + GLE treatment recorded the least. Additionally, LA-treated samples revealed lower scores for odor and taste than GLE and LA + GLE after treatment and throughout storage. The decline in fruit smell and taste with prolonged storage can be explained by the changes that occurred with the advancement of fruits’ ripening e.g., transformation of sucrose into reducing sugars, the decomposition of the compounds of odor and taste, and the formation of new metabolic products [4]. Moreover, Seddiek et al. [16] attributed the decrease in apples’ taste and odor during storage to the microbial growth and metabolic activity accompanied by respiration.

Moreover, the results presented in Table 6 revealed a decrease in the texture scores for all Barhi date samples with extended storage. Generally, the control showed the greatest drop (6.82), whereas LA + GLE treatment revealed the least (7.13) after three and five weeks of storage, respectively. Kumar et al. [30] and Gull et al. [32] illustrated the drop in texture scores of stored fruits by the loss of moisture content, the decomposition of pectic compounds, and the advancement of ripening and senescence processes. It is worth noting that the results for firmness recorded by panelists conformed with those obtained by the instrument (Section 3.5).

Likewise, all samples exhibited a gradual decline in overall acceptability during storage. The control sample exhibited the lowest score (6.83) after 3 weeks. On the contrary, the LA + GLE treatment showed better storage stability during storage than other treatments, with a score of 7.15 after 5 weeks. It could be suggested that the synergistic effect of LA + GLE may be responsible for maintaining quality and prolonging the Barhi date fruits’ shelf life through reducing microbial growth, decreasing the respiration rate, reducing enzymatic browning and retarding pectic substance degradation [4,16]. Our results were compatible with the findings of Seddiek et al. [16] and Nair et al. [38], who reported that LA and natural plant extracts maintained the sensory quality of apple and bell pepper, respectively, during storage.

### 3.7. Microbiological Quality

Microbial decomposition represents a principal factor causing quality loss and shortening the shelf life of fresh food products. As fruits typically have lower pH values with higher sugar contents, yeasts and molds are mainly expected to cause spoilage compared to bacterial strains [41]. Changes in yeast and mold numbers in stored Barhi dates with different treatments are given in Table 7. On day 0 and by the end of the first week of storage, the samples treated with natural preservatives showed no microbial growth, while the control contained 2.07 and 2.89 log CFU/g, respectively. With the progression of storage time, all samples revealed a gradual increase in yeast and mold counts, with the greatest increase achieved by the control and the lowest increase obtained by LA + GLE treatment. At the end of the experiment, the control exhibited the largest number of yeasts and molds (4.19 log CFU/g), while LA + GLE treatment showed the lowest (2.00 log CFU/g). Among all treatments, the control sample showed the shortest shelf life (3 weeks), whereas LA + GLE-treated sample revealed the longest (5 weeks).

It could be suggested that the reduced counts of yeasts and molds in treated fruits may be due to the synergistic effect of LA and GLE. The antifungal activities of LA and GLE were confirmed by several studies [6,7,15,16,42,43]. LA inhibits fungal growth probably through cell membrane disruption, proton and anion accumulation, acidification of intracellular components, and depletion of ATP (an organic compound providing energy necessary for cellular growth) [42,43]. Likewise, the antifungal effects of GLE are due to the presence of bioactive compounds with antifungal effects, e.g., gallic acid [8,29], chlorogenic acid, kaempferol, morin, rutin, avicularin, quercitrin, isoquercitrin, and quercetin, which can cause degradation of the fungal cell membrane and inhibition of cell growth [8]. Generally, all Barhi date samples in the present study showed lower yeast and mold counts than 6.0 log CFU/g (the maximum limit allowed for stored fruit quality) [16]. Our results were consistent with the findings of Seddiek et al. [16] and Alqahtani et al. [4], who reported that LA and natural plant extracts decreased the yeast and mold counts of apple fruits and Khalal Barhi dates, respectively, throughout storage.

## 4. Conclusions

The present study revealed that disinfection treatment with natural preservatives extended the shelf life of Khalal Barhi dates. The treatments increased TSS and prevented moisture loss, color changes, and firmness deterioration throughout storage. Additionally, the dip treatments reduced the yeasts and molds and preserved the sensorial properties of the Barhi dates during storage. Further, the GLE- and LA + GLE-treated samples exhibited higher TPC and antioxidant activity and preserved higher values with the progression of storage. In general, the LA + GLE treatment achieved the best storage stability among all treatments. It could be suggested that LA + GLE disinfection treatment may be a promising technique to maintain quality and extend Khalal Barhi dates’ shelf life. However, further studies are still needed for commercial application.

## Figures and Tables

**Table 1:** HPLC analysis of polyphenolic compounds in guava leaf extract.

Polyphenolic Compound	Conc. (mg/g)
Gallic acid	14.856 ± 0.32
Chlorogenic acid	0.507 ± 0.06
Catechin	0.898 ± 0.07
Methyl gallate	0.064 ± 0.01
Caffeic acid	0.123 ± 0.02
Syringic acid	0.087 ± 0.01
Pyrocatechol	7.589 ± 0.25
Rutin	0.096 ± 0.01
Ellagic acid	18.403 ± 0.41
Coumaric acid	ND
Vanillin	0.360 ± 0.04
Ferulic acid	1.083 ± 0.08
Naringenin	2.477 ± 0.09
Daidzein	ND
Quercetin	0.110 ± 0.02
Cinnamic acid	0.004 ± 0.001
Apigenin	ND
Kaempferol	ND
Hesperetin	ND

The results are presented as means ± standard deviation. ND: not detected.

**Table 2:** Changes in physicochemical qualities of fresh Barhi dates treated with natural preservatives during cold storage (4 ± 1 °C).

Treatments	Storage Period (Week)					
	0	1	2	3	4	5
	Moisture content (%)					
Control	68.39 ± 0.92 ^Aa^	67.11 ± 0.76 ^Aa^	65.30 ± 1.57 ^Ab^	62.21 ± 0.86 ^Bc^	-	-
LA	68.42 ± 1.24 ^Aa^	67.40 ± 1.15 ^Aab^	65.64 ± 1.42 ^Abc^	64.19 ± 1.13 ^ABcd^	62.28 ± 1.05 ^Ad^	-
GLE	68.44 ± 0.77 ^Aa^	67.53 ± 0.86 ^Aab^	66.32 ± 1.46 ^Abc^	64.72 ± 0.88 ^Ac^	62.54 ± 0.94 ^Ad^	-
LA + GLE	68.38 ± 1.31 ^Aa^	67.58 ± 0.84 ^Aab^	66.43 ± 1.68 ^Aab^	65.21 ± 1.42 ^Abc^	63.73 ± 1.24 ^Ac^	63.04 ± 1.14 ^Ac^
	Total soluble solids (%)					
Control	27.15 ± 0.63 ^Ad^	29.39 ± 0.57 ^Ac^	32.65 ± 0.54 ^Ab^	33.77 ± 0.60 ^Aa^	-	-
LA	27.16 ± 0.38 ^Ad^	29.17 ± 0.55 ^Ac^	31.76 ± 0.70 ^Ab^	32.60 ± 0.42 ^Ba^	33.14 ± 0.39 ^Aa^	-
GLE	27.19 ± 0.64 ^Ad^	28.87 ± 0.68 ^Ac^	30.23 ± 0.72 ^Bb^	31.51 ± 0.47 ^Ca^	32.11 ± 0.62 ^Ba^	-
LA + GLE	27.17 ± 0.83 ^Ad^	28.74 ± 0.60 ^Ac^	29.92 ± 0.86 ^Bb^	31.08 ± 0.56 ^Ca^	31.62 ± 0.61 ^Ba^	32.02 ± 0.24 ^Aa^
	pH					
Control	6.25 ± 0.19 ^Aa^	6.03 ± 0.54 ^Aab^	5.60 ± 0.14 ^Ab^	4.93 ± 0.23 ^Bc^	-	-
LA	6.07 ± 0.34 ^Aa^	5.89 ± 0.35 ^Aa^	5.64 ± 0.42 ^Aab^	5.23 ± 0.20 ^ABbc^	5.07 ± 0.18 ^Ac^	-
GLE	6.24 ± 0.47 ^Aa^	6.07 ± 0.27 ^Aa^	5.78 ± 0.33 ^Aab^	5.58 ± 0.56 ^ABab^	5.19 ± 0.22 ^Ab^	-
LA + GLE	6.17 ± 0.33 ^Aa^	6.05 ± 0.51 ^Aa^	5.89 ± 0.09 ^Aab^	5.65 ± 0.32 ^Aabc^	5.38 ± 0.30 ^Abc^	5.21 ± 0.13 ^Ac^
	Titratable acidity (%)					
Control	0.13 ± 0.05 ^Ac^	0.17 ± 0.04 ^Abc^	0.22 ± 0.03 ^Ab^	0.28 ± 0.04 ^Aa^	-	-
LA	0.15 ± 0.06 ^Ab^	0.17 ± 0.06 ^Ab^	0.20 ± 0.07 ^Aab^	0.24 ± 0.03 ^Aab^	0.27 ± 0.05 ^Aa^	-
GLE	0.13 ± 0.04 ^Ac^	0.15 ± 0.04 ^Abc^	0.19 ± 0.06 ^Aabc^	0.22 ± 0.06 ^Aab^	0.25 ± 0.02 ^Aa^	-
LA + GLE	0.14 ± 0.01 ^Ab^	0.15 ± 0.01 ^Ab^	0.18 ± 0.03 ^Aab^	0.21 ± 0.03 ^Aa^	0.23 ± 0.05 ^Aa^	0.24 ± 0.04 ^Aa^

The results are presented as the means ± standard deviation; *n* = 3. Means followed by different superscript letters within each column (upper case) or row (lower case) are significantly different (*p* < 0.05). LA, lactic acid; GLE, guava leaf extract; -, not determined due to sensorial rejection or spoilage.

**Table 3:** Changes in total phenolic content and antioxidant activity in fresh Barhi dates treated with natural preservatives during cold storage (4 ± 1 °C).

Treatments	Storage Period (Week)					
	0	1	2	3	4	5
	Total phenolic content (mg GAE/g)					
Control	8.18 ± 0.32 ^Ba^	6.21 ± 0.38 ^Bb^	4.58 ± 0.41 ^Bc^	3.27 ± 0.55 ^Cd^	-	-
LA	8.19 ± 0.29 ^Ba^	6.58 ± 0.24 ^Bb^	5.15 ± 0.34 ^Bc^	4.54 ± 0.32 ^Bd^	4.07 ± 0.36 ^Bd^	-
GLE	9.91 ± 0.29 ^Aa^	8.00 ± 0.32 ^Ab^	6.74 ± 0.42 ^Ac^	5.07 ± 0.28 ^ABd^	4.35 ± 0.29 ^Be^	-
LA + GLE	9.62 ± 0.32 ^Aa^	8.61 ± 0.51 ^Ab^	7.33 ± 0.28 ^Ac^	5.62 ± 0.43 ^Ad^	5.04 ± 0.41 ^Ade^	4.42 ± 0.35 ^Ae^
	DPPH scavenging (%)					
Control	44.28 ± 0.82 ^Ba^	36.35 ± 0.64 ^Cb^	27.51 ± 0.87 ^Cc^	23.76 ± 0.62 ^Dd^	-	-
LA	44.29 ± 0.77 ^Ba^	37.14 ± 0.92 ^Cb^	31.63 ± 0.90 ^Bc^	27.20 ± 0.75 ^Cd^	24.46 ± 0.88 ^Ce^	-
GLE	48.18 ± 1.23 ^Aa^	40.38 ± 0.87 ^Bb^	33.64 ± 1.34 ^Ac^	29.92 ± 1.45 ^Bd^	26.13 ± 0.95 ^Be^	-
LA + GLE	47.82 ± 1.13 ^Aa^	42.35 ± 0.93 ^Ab^	34.56 ± 0.82 ^Ac^	32.35 ± 1.22 ^Ad^	30.19 ± 1.19 ^Ae^	26.87 ± 1.22 ^Af^

The results are presented as the means ± standard deviation; *n* = 3. Means followed by different superscript letters within each column (upper case) or row (lower case) are significantly different (*p* < 0.05). LA, lactic acid; GLE, guava leaf extract; -, not determined due to sensorial rejection or spoilage.

**Table 4:** Changes in color parameters of fresh Barhi dates treated with natural preservatives during cold storage (4 ± 1 °C).

Treatments	Storage Period (Week)					
	0	1	2	3	4	5
	L*					
Control	55.21 ± 1.25 ^Aa^	50.17 ± 0.75 ^Bb^	44.61 ± 1.34 ^Bc^	41.56 ± 0.75 ^Cd^	-	-
LA	55.83 ± 0.77 ^Aa^	51.35 ± 1.09 ^ABb^	47.94 ± 1.30 ^Ac^	43.23 ± 0.89 ^Bd^	41.72 ± 1.19 ^Bd^	-
GLE	54.15 ± 1.06 ^Aa^	51.64 ± 1.30 ^ABb^	48.50 ± 1.25 ^Ac^	45.75 ± 1.09 ^Ad^	42.00 ± 1.36 ^Be^	-
LA + GLE	55.00 ± 0.96 ^Aa^	52.90 ± 1.12 ^Ab^	50.14 ± 1.43 ^Ac^	47.13 ± 0.77 ^Ad^	44.65 ± 1.28 ^Ae^	42.88 ± 1.12 ^Ae^
	a*					
Control	1.43 ± 0.08 ^Ad^	1.64 ± 0.07 ^Ac^	1.92 ± 0.06 ^Ab^	2.13 ± 0.04 ^Aa^	-	-
LA	1.44 ± 0.05 ^Ae^	1.55 ± 0.06 ^Ad^	1.83 ± 0.09 ^Ac^	2.00 ± 0.06 ^Bb^	2.12 ± 0.07 ^Aa^	-
GLE	1.35 ± 0.08 ^Ad^	1.41 ± 0.05 ^Bd^	1.59 ± 0.04 ^Bc^	1.85 ± 0.07 ^Cb^	2.07 ± 0.04 ^Aa^	-
LA + GLE	1.32 ± 0.06 ^Ae^	1.36 ± 0.04 ^Be^	1.51 ± 0.05 ^Bd^	1.73 ± 0.04 ^Dc^	1.84 ± 0.05 ^Bb^	2.00 ± 0.08 ^Aa^
	b*					
Control	36.34 ± 0.88 ^Aa^	31.08 ± 1.11 ^Ab^	26.52 ± 0.91 ^Bc^	22.05 ± 0.81 ^Cd^	-	-
LA	36.71 ± 1.19 ^Aa^	32.15 ± 1.35 ^Ab^	29.13 ± 0.86 ^Ac^	25.22 ± 0.68 ^Bd^	22.34 ± 0.90 ^Be^	-
GLE	35.76 ± 1.31 ^Aa^	32.62 ± 0.84 ^Ab^	30.07 ± 0.79 ^Ac^	26.36 ± 1.16 ^ABd^	23.70 ± 0.83 ^Be^	-
LA + GLE	35.14 ± 1.13 ^Aa^	33.05 ± 0.87 ^Ab^	30.59 ± 1.05 ^Ac^	27.54 ± 0.82 ^Ad^	25.81 ± 0.77 ^Ae^	23.87 ± 0.58 ^Af^

The results are presented as the means ± standard deviation; *n* = 3. Means followed by different superscript letters within each column (upper case) or row (lower case) are significantly different (*p* < 0.05). LA, lactic acid; GLE, guava leaf extract; -, not determined due to sensorial rejection or spoilage.

**Table 5:** Changes in firmness of fresh Barhi dates treated with natural preservatives during cold storage (4 ± 1 °C).

Treatments	Storage Period (Week)					
	0	1	2	3	4	5
	Firmness					
Control	1049 ± 6 ^Aa^	729 ± 5 ^Cb^	241 ± 7 ^Dc^	80 ± 5 ^Dd^	-	-
LA	1050 ± 7 ^Aa^	738 ± 8 ^Cb^	285 ± 5 ^Cc^	97 ± 9 ^Cd^	82 ± 3 ^Ce^	-
GLE	1046 ± 6 ^Aa^	753 ± 4 ^Bb^	322 ± 3 ^Bc^	139 ± 2 ^Bd^	86 ± 2 ^Be^	-
LA + GLE	1057 ± 6 ^Aa^	765 ± 3 ^Ab^	347 ± 6 ^Ac^	161 ± 4 ^Ad^	103 ± 1 ^Ae^	87 ± 3 ^Af^

The results are presented as the means ± standard deviation; *n* = 3. Means followed by different superscript letters within each column (upper case) or row (lower case) are significantly different (*p* < 0.05). LA, lactic acid; GLE, guava leaf extract; -, not determined due to sensorial rejection or spoilage.

**Table 6:** Changes in sensory evaluation scores for fresh Barhi dates treated with natural preservatives during cold storage (4 ± 1 °C).

Treatments	Storage Period (Week)					
	0	1	2	3	4	5
	Appearance					
Control	8.20 ± 0.59 ^Aa^	7.61 ± 0.55 ^Aab^	7.23 ± 0.48 ^Ab^	6.17 ± 0.77 ^Ac^	-	-
LA	8.21 ± 0.62 ^Aa^	7.85 ± 0.74 ^Aa^	7.49 ± 0.57 ^Aab^	6.78 ± 0.36 ^Abc^	6.29 ± 0.41 ^Bc^	-
GLE	8.19 ± 0.26 ^Aa^	8.00 ± 0.21 ^Aa^	7.74 ± 0.53 ^Aa^	7.33 ± 0.75 ^Aab^	6.75 ± 0.55 ^ABb^	-
LA + GLE	8.14 ± 0.53 ^Aa^	8.06 ± 0.82 ^Aa^	7.82 ± 0.20 ^Aab^	7.54 ± 0.91 ^Aab^	7.12 ± 0.42 ^Aab^	6.84 ± 0.53 ^Ab^
	Odor					
Control	8.32 ± 0.35 ^Aa^	8.00 ± 0.51 ^Aab^	7.53 ± 0.35 ^Abc^	7.22 ± 0.30 ^Ac^	-	-
LA	8.17 ± 0.42 ^Aa^	8.03 ± 0.36 ^Aab^	7.70 ± 0.47 ^Aab^	7.41 ± 0.54 ^Aab^	7.26 ± 0.52 ^Ab^	-
GLE	8.38 ± 0.51 ^Aa^	8.16 ± 0.83 ^Aa^	7.85 ± 0.80 ^Aa^	7.56 ± 0.83 ^Aa^	7.29 ± 0.47 ^Aa^	-
LA + GLE	8.26 ± 0.48 ^Aa^	8.18 ± 0.65 ^Aa^	8.09 ± 0.19 ^Aa^	7.73 ± 0.60 ^Aa^	7.57 ± 0.39 ^Aa^	7.32 ± 0.45 ^Aa^
	Taste					
Control	8.30 ± 0.18 ^Aa^	8.02 ± 0.20 ^Aa^	7.59 ± 0.85 ^Aab^	7.11 ± 0.36 ^Ab^	-	-
LA	8.19 ± 0.59 ^Aa^	8.05 ± 0.36 ^Aab^	7.78 ± 0.54 ^Aab^	7.50 ± 0.45 ^Aab^	7.17 ± 0.61 ^Ab^	-
GLE	8.33 ± 0.55 ^Aa^	8.20 ± 0.61 ^Aab^	8.01 ± 0.37 ^Aab^	7.69 ± 0.39 ^Aab^	7.23 ± 0.80 ^Ab^	-
LA + GLE	8.24 ± 0.52 ^Aa^	8.19 ± 0.40 ^Aa^	8.08 ± 0.82 ^Aa^	7.94 ± 0.52 ^Aa^	7.62 ± 0.74 ^Aa^	7.32 ± 0.63 ^Aa^
	Texture					
Control	8.44 ± 0.31 ^Aa^	8.10 ± 0.29 ^Aab^	7.49 ± 0.85 ^Abc^	6.82 ± 0.77 ^Ac^	-	-
LA	8.41 ± 0.52 ^Aa^	8.28 ± 0.30 ^Aa^	7.75 ± 0.51 ^Aab^	7.37 ± 0.65 ^Ab^	7.04 ± 0.28 ^Ab^	-
GLE	8.42 ± 0.49 ^Aa^	8.34 ± 0.37 ^Aab^	8.03 ± 0.29 ^Aab^	7.69 ± 0.36 ^Abc^	7.10 ± 0.44 ^Ac^	-
LA + GLE	8.45 ± 0.32 ^Aa^	8.39 ± 0.52 ^Aa^	8.14 ± 0.81 ^Aa^	7.85 ± 0.54 ^Aab^	7.51 ± 0.42 ^Aab^	7.13 ± 0.27 ^Ab^
	Overall acceptability					
Control	8.32 ± 0.61 ^Aa^	7.93 ± 0.57 ^Aab^	7.46 ± 0.34 ^Abc^	6.83 ± 0.47 ^Ac^	-	-
LA	8.25 ± 0.58 ^Aa^	8.05 ± 0.64 ^Aab^	7.68 ± 0.60 ^Aabc^	7.27 ± 0.82 ^Abc^	6.94 ± 0.56 ^Ac^	-
GLE	8.33 ± 0.91 ^Aa^	8.18 ± 0.38 ^Aa^	7.91 ± 0.71 ^Aab^	7.56 ± 0.91 ^Aab^	7.09 ± 0.39 ^Ab^	-
LA + GLE	8.27 ± 0.28 ^Aa^	8.21 ± 0.55 ^Aa^	8.03 ± 0.55 ^Aab^	7.77 ± 0.72 ^Aab^	7.46 ± 0.41 ^Aab^	7.15 ± 0.62 ^Ab^

The results are presented as the means ± standard deviation; *n* = 3. Means followed by different superscript letters within each column (upper case) or row (lower case) are significantly different (*p* < 0.05). LA, lactic acid; GLE, guava leaf extract; -, not determined due to sensorial rejection or spoilage.

**Table 7:** Changes in yeast and mold counts (log CFU/g) of fresh Barhi dates treated with natural preservatives during cold storage (4 ± 1 °C).

Treatments	Storage Period (Week)					
	0	1	2	3	4	5
Control	2.07 ± 0.23 ^Ad^	2.89 ± 0.30 ^Ac^	3.38 ± 0.19 ^Ab^	4.19 ± 0.25 ^Aa^	-	-
LA	n.d	n.d	0.58 ± 0.24 ^Bc^	1.67 ± 0.26 ^Bb^	2.14 ± 0.23 ^Aa^	-
GLE	n.d	n.d	0.55 ± 0.18 ^Bc^	1.62 ± 0.16 ^Bb^	2.11 ± 0.16 ^Aa^	-
LA + GLE	n.d	n.d	0.46 ± 0.17 ^Bc^	1.25 ± 0.22 ^Bb^	1.82 ± 0.20 ^Aa^	2.00 ± 0.14 ^Aa^

The results are presented as the means ± standard deviation; *n* = 3. Means followed by different superscript letters within each column (upper case) or row (lower case) are significantly different (*p* < 0.05). LA, lactic acid; GLE, guava leaf extract; n.d, not detected; -, not determined due to sensorial rejection or spoilage.

## Data Availability

The data presented in this study are available on request from the corresponding author.

## Supplementary Materials

The following supporting information can be downloaded at: https://www.mdpi.com/article/10.3390/foods12112115/s1, Table S1: HPLC analysis of polyphenolic compounds in the standards; Figure S1: HPLC chromatogram of guava leaf extract.
