# Supplementary material for: Quality Attributes of Cold-Stored Khalal Barhi Dates Treated with Guava Leaf Extract and/or Lactic Acid as Natural Preservatives
Source: Foods. 2023 May 24;12(11):2115. doi: 10.3390/foods12112115 (PMC10252430; doi:10.3390/foods12112115)
Supplement: Supplementary file 1 [file foods-12-02115-s001.zip › foods-2380446-supplementary.pdf]

Supplementary file
Manuscript ID: foods-2380446

Table S1. HPLC analysis of polyphenolic compounds in the standards.

Polyphenolic compound	Conc. (µg/ml)
Gallic acid	15
Chlorogenic acid	50
Catechin	75
Methyl gallate	15
Caffeic acid	18
Syringic acid	17.2
Pyro catechol	40
Rutin	26
Ellagic acid	120
Coumaric acid	20
Vanillin	12.9
Ferulic acid	20
Naringenin	30
Daidzein	35
Quercetin	40
Cinnamic acid	10
Apigenin	50
Kaempferol	20
Hesperetin	20

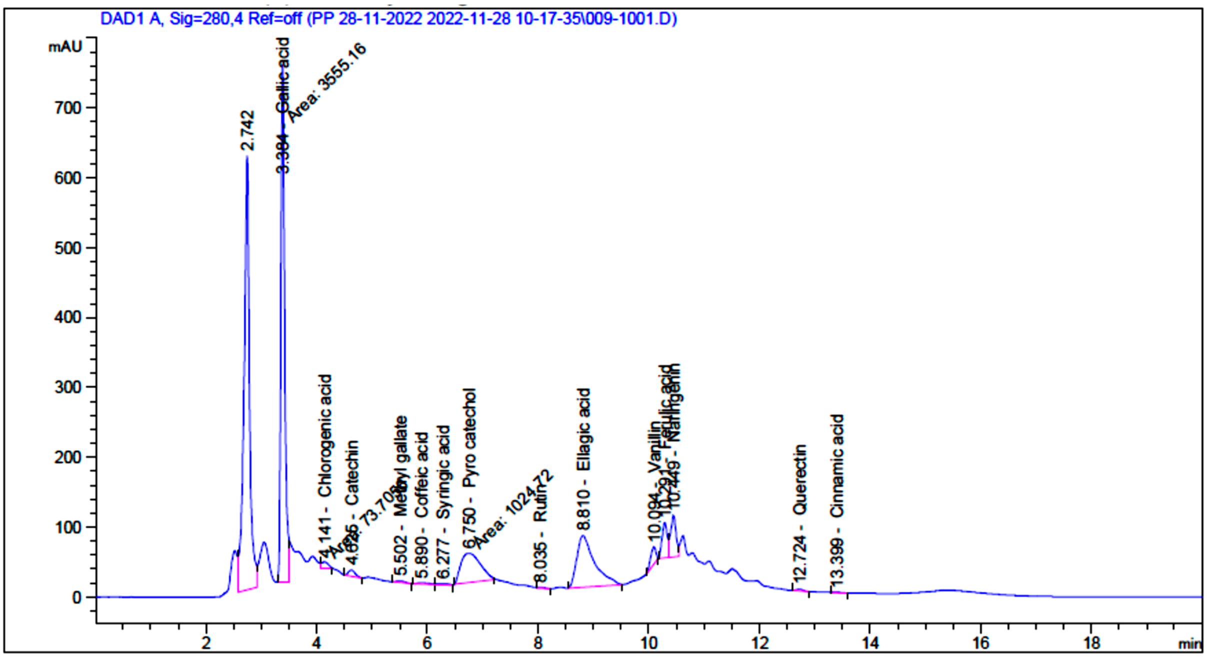

Figure S1. HPLC chromatogram of guava leaf extract.
